# Engineered VP1 mRNA Vaccine Induces Immunity and Complete Protection Against Feline Calicivirus in Cats

**DOI:** 10.1155/tbed/9499266

**Published:** 2026-02-06

**Authors:** Meng-Di Zhang, Zhuo-Fang Xie, Xin-Hong Li, Wei Huang, Qian-Yu Qian, Hong-Tao Cao, Ji-Wei Liu, Ya-Qing Zhang, Bin Wang, Yu Qin, Fu-Shan Shi, Jian-Bin Tang, Yao-Wei Huang, Yong-Le Yang

**Affiliations:** ^1^ Guangdong Laboratory for Lingnan Modern Agriculture, College of Veterinary Medicine, South China Agricultural University, Guangzhou, 510642, Guangdong, China, scau.edu.cn; ^2^ Xianghu Laboratory, Hangzhou, 311231, Zhejiang, China; ^3^ College of Life Sciences, Zhejiang Chinese Medical University, Hangzhou, 310053, Zhejiang, China, zcmu.edu.cn; ^4^ Zhejiang Key Laboratory of Smart Biomaterials, College of Chemical and Biological Engineering, Zhejiang University, Hangzhou, 310058, Zhejiang, China, zju.edu.cn; ^5^ Department of Veterinary Medicine, Zhejiang University, Hangzhou, 310058, Zhejiang, China, zju.edu.cn; ^6^ State Key Laboratory of Animal Disease Control and Prevention, South China Agricultural University, Guangzhou, 510642, Guangdong, China, scau.edu.cn

**Keywords:** feline calicivirus, immune response, mRNA vaccine, VP1 antigen

## Abstract

Feline calicivirus (FCV) is a major pathogen of upper respiratory tract diseases in cats, posing a significant threat to feline health. While current FCV preventive measures rely primarily on traditional vaccines, messenger RNA (mRNA) vaccines have emerged as a promising alternative, offering high efficacy, safety, rapid clinical development, and potential for fast, cost‐efficient production. In this study, we designed a modified nucleotide sequence with a 124‐amino acid deletion at a position in the region of the FCV‐VP1 protein as an immunogen. The plasmid encoding the codon‐optimized VP1 sequence was constructed, and VP1‐mRNA was generated by in vitro transcription (IVT) and capping. After transfection into BHK‐21 cells, immunofluorescence assay (IFA) and WB confirmed successful FCV‐VP1 expression. Subsequently, the mRNA was encapsulated into lipid nanoparticles (LNPs) to prepare the LNP‐VP1‐mRNA vaccine. Characterization analysis revealed a uniform particle size distribution (polydispersity index [PDI] = 0.169) and a stable surface charge (zeta potential = −1.67 mV). A prime‐boost immunization strategy was employed, which involved two intramuscular injections to immunize BALB/c mice or cats with the LNP‐VP1‐mRNA vaccine. ELISA analysis demonstrated that the vaccine elicited elevated levels of anti‐FCV IgG and neutralizing antibodies in a dose‐dependent manner, accompanied by the secretion of cytokines including IFN‐β, IFN‐γ, IL‐4, and IL‐6. Importantly, the VP1 mRNA vaccine provided complete protection against FCV challenge in cats, without the typical clinical signs and with a 100% survival rate. Our results indicate that the LNP‐VP1‐mRNA vaccine is a promising candidate for combating FCV infection.

## 1. Introduction

Feline calicivirus (FCV) disease (FCVD) is an acute, contact‐transmissible infectious disease that mainly manifests as an upper respiratory tract infection caused by FCV [1]. FCV can induce severe disease in domestic cats and large wild felines such as tigers and lions, and there have also been reports of dog infections [2–4]. Pet cats have become an important component of human life, and contagious infectious diseases present a significant risk to feline health. FCV infection in young cats is mainly characterized by oral ulcers, conjunctivitis, and runny nose, while adult cats primarily manifest symptoms such as feline chronic gingivostomatitis (FCGs) [5]. Therefore, effective prevention and control strategies are essential, and the FCVD vaccine has become one of the core vaccines in the feline immunization regimen [6]. In recent years, highly pathogenic FCV strains capable of virulent systemic disease have been reported globally, with significant mortality rates observed in Europe, Asia, and beyond [7–10]. FCV is highly prevalent and contagious, and vaccination remains the principal means of control [11]. FCV vaccines are classified into attenuated live vaccines and inactivated vaccines, though research shows that commercially available vaccines exhibit low neutralization titers [12–14]. Rehabilitated cats continue to excrete pathogens for an extended period, posing a significant threat to the health of pet cats and other feline species [15]. The development of novel, safe, and efficacious FCV vaccines is urgently required to mitigate health risks in domestic cats.

The FCV viral genome is a positive‐sense, single‐stranded RNA molecule of ~7.7 kilobases (kb) [16, 17]. The genome contains three open reading frames (ORFs), of which ORF1 encodes nonstructural proteins, ORF2 encodes the capsid protein VP1, and ORF3 encodes the minor structural protein VP2 [18–20]. The ORF2 gene encoding the capsid protein precursor was cleaved by the ORF1 protease to produce LC (leader peptide), a 124‐amino acid peptide, and mature VP1. Among these, the VP1 protein represents the primary antigenic determinant of FCV, and its protein consists of six structural regions (A–F). While the N‐terminal A region (1–124 aa) is excised during capsid maturation, the rest of the regions contain most of the neutralizing epitopes, which are the key targets of the antibody response [21–23]. Therefore, VP1 was selected as the target antigen for messenger RNA (mRNA) vaccine in this study.

Nucleoside‐modified mRNA vaccines are a new type of vaccine technology that has emerged in recent years. With the rapid advancement of RNA biology research, the mRNA stability and delivery efficiency have been greatly improved. mRNA vaccine technology has been applied in the development of vaccines targeting multiple infectious diseases, including influenza virus [24], SARS‐CoV‐2 [25], dengue virus [26], rabies virus, and porcine epidemic diarrhea virus [27, 28]. In contrast to other vaccine platforms, these vaccines contain nucleoside‐modified mRNA encoding pathogen antigens complexed within lipid nanoparticles (LNPs). The complex protects the mRNA and allows for efficient uptake and translation of the encoded pathogen antigens by host cells to elicit both cellular and humoral immune responses [29, 30].

In this study, the mRNA molecule of calicivirus VP1 was designed, constructed, and identified, and the candidate mRNA vaccine VP1‐mRNA encapsulated by LNPs was further prepared. The immunogenicity of the candidate vaccine was evaluated in mice, and its protective efficacy was further assessed in cats.

## 2. Materials and Methods

### 2.1. Cells, Viruses, and Animals

Both the BHK‐21 and CRFK cells were cultured and propagated in Dulbecco’s modified Eagle medium (DMEM; Thermo Fisher, USA) with 10% fetal bovine serum (FBS; Thermo Fisher, USA) and 1% penicillin (100 U/mL)—streptomycin (100 μg/mL) (Thermo Fisher, USA). All cells were grown at 37°C in a humidified 5% CO_2_ atmosphere. The FCV strain was provided by Professor Fu‐Shan Shi from Zhejiang University [31]. Female BALB/c mice aged 6–8 weeks were purchased from Hangzhou Hangs Biotech Co., Ltd. (Hangzhou, China). Healthy Chinese domestic cats aged around 2–3 months (nasal, ocular, and anal swabs tested negative for FCV nucleic acids; serum FCV neutralizing titers no higher than 1:4) were sourced from Qindao Yibang Bioengineering Co., Ltd. (Shandong, China).

### 2.2. Plasmid Construction

The VP1 gene from the FCV strain supplied by Professor Shifu Shan was selected as the target antigen for the mRNA vaccine, as the VP1 protein serves as the major capsid protein of FCV and contains specific antigenic neutralization sites. To enhance protein expression, improve immune recognition, and reduce potential immunogenicity, a 124‐amino acid deletion was introduced in the N‐terminal region, corresponding to the leader sequence cleaved during capsid maturation. This deletion removes the leader peptide, which is not involved in antigenicity, while preserving the immunodominant neutralizing epitopes in the remaining VP1 protein. Additionally, codon optimization was performed to further enhance protein expression and reduce immune escape, ensuring the correct presentation of neutralizing epitopes. The sequence was embedded into an mRNA construct containing the 5^′^ and 3^′^ untranslated regions (UTRs) of human α‐globin. Additionally, the signal peptide (SP) at the 5^′^ end of the ORF, a 5^′^ cap, and a 100‐nucleotide poly(A) tail at the 3^′^ end were added. The complete VP1 mRNA sequence is provided in Supporting Information 1. The whole genome was synthesized and inserted into the pUC57 vector by Nanjing Kingsley Biotechnology (Nanjing, China) to obtain the pUC57‐VP1 recombinant plasmid.

### 2.3. In Vitro Transcription (IVT) and mRNA Construction

For the IVT mRNA production, plasmids were linearized with *Bsa* I (NEB; Beijing, China) and subsequently purified. The linearized plasmid served as a template for the IVT reaction, which was conducted using the T7 High Yield RNA Transcription Kit (Nearshore Protein, Suzhou, China) following the manufacturer’s instructions. The RNA was capped using the enzymatic reaction Cap 1 Capping System kit with 7‐methylguanosine cap structure (m7Gppp, Cap0) according to the manufacturer’s instructions (Nearshore Protein; Suzhou, China). The capped mRNA products were purified by DNase I digestion, followed by precipitation with Trizol reagent and washing with 75% ethanol. All mRNAs were analyzed by agarose gel electrophoresis, and concentrations were determined by measuring the absorbance at 260 nm. The purity of mRNA was determined by capillary electrophoresis using a 2100 Bioanalyzer, and the samples were stored at −80°C until further use.

### 2.4. Immunofluorescence Assay (IFA) for FCV VP1 Protein Expression

To validate VP1‐mRNA expression, we performed the following experiments. In brief, BHK‐21 cells were plated into a 48‐well plate at a density of 2 × 10^5^ cells per well. After culturing for 20 h, 2 μg mRNA was transfected into cells using DMRIE‐C transfection reagent (Invitrogen, USA) according to the manufacturer’s instructions, with untreated cells serving as a control to assess the background signal of the anti‐VP1 antibody. After transfection for 24 h, cells were fixed with 4% paraformaldehyde for 15 min, and then cells were permeabilized with 0.1% Triton. After three washes with PBS, the cells were incubated with an anti‐FCV‐VP1 rabbit polyclonal antibody (generated by immunizing New Zealand white rabbits with synthetic peptides corresponding to VP1 segments STLPETGARGGNHPC and TATLDGDNNNKINPC; 1:1000) for 1.5 h at 37°C. Afterwards, the cells were incubated with an Alexa‐Fluor‐488‐conjugated goat anti‐rabbit IgG (Beyotime, 1:5000) for 1 h at 37°C. Finally, the nuclei of the cells were stained with diamidino‐2‐phenylindole (DAPI; Solarbio, 1:1000) staining solution, which was used to stain the nuclei of the cells, and were observed under a fluorescence microscope (Zeiss, Oberkochen, Germany).

### 2.5. Western Blot Assay for FCV VP1 Protein Expression

BHK‐21 cells were plated into a 6‐well plate at a density of 2 × 10^5^ cells per well. Six microgram mRNA was transfected into the cells using DMRIE‐C transfection reagent (Invitrogen, USA) according to the manufacturer’s instructions. The mixtures were then added to the cell culture medium and incubated for 24 h. The cell lysates were then collected, and the expression of VP1 protein was analyzed by Western blot. Samples were mixed with 5× loading buffer, boiled for 10 min, separated by sodium dodecyl sulfate‐polyacrylamide gel electrophoresis (SDS‐PAGE), and electrotransferred onto 0.22‐μm‐pore‐size polyvinylidene difluoride (PVDF) membranes using a Mini Trans‐Blot Cell transfer apparatus (Bio‐Rad, USA). Membranes were blocked with 5% skim milk for 1 h at room temperature and washed three times with phosphate‐buffered saline containing 0.1% Tween 20 (PBST, 0.1% Tween 20). For VP1 protein detection, membranes were incubated with a rabbit polyclonal anti‐FCV‐VP1 antibody (1:1000) for 1.5 h at room temperature, followed by incubation with a goat anti‐mouse horseradish peroxidase (HRP)‐conjugated secondary antibody (1:5000) for 1 h at room temperature. Finally, the protein bands were visualized using a ChemiDoc Image Lab chemiluminescent imaging system (Bio‐Rad, USA).

### 2.6. Preparation of LNP‐VP1‐mRNA Vaccine

For the preparation of the LNP‐VP1‐mRNA vaccine, VP1‐mRNA was mixed in 20 mM sodium acetate buffer, while lipids were dissolved in ethanol at a molar ratio of 50:10:38.5:1.5 (SM‐102, DSPC, PEG‐lipid, and cholesterol) [29, 32]. The LNP solution and the mRNA solution were combined at a 1:3 volume ratio and vortexed for 20 s, then allowed to stand at room temperature for 10 min, resulting in the formation of LNP‐VP1‐mRNA vaccine particles. LNP‐encapsulated mRNA vaccine was analyzed for particle size, zeta potential, and polydispersity index (PDI) using a Zetasizer Ultra instrument (Malvern Panalytical; Malvern, UK). The LNP‐VP1‐mRNA vaccine was formulated with 5% sucrose and stored at ‐80°C.

### 2.7. Ethics Statement

The experimental protocol was reviewed and approved by the Scientific Ethics Committee of Guangdong Provincial Laboratory Animal Monitoring Institute with the Approval Number, I‐IACUC2024101. The immunization of cats was conducted at the Biosafety Level 2 of Qindao Yibang Bioengineering Co., Ltd. (Shandong, China). Considering animal welfare, all the cats were treated using symptomatic treatment methods.

### 2.8. Mice Immunization Experiments and Serum Collection

Female BALB/c mice aged 6–8 weeks were randomly assigned to three groups: the LNP‐VP1‐mRNA vaccine group, the positive vaccine group, and the control group (*n* = 4). Mice in all groups received intramuscular injections at weeks 0 and 4. The LNP‐VP1‐mRNA vaccine was administered at 5 μg per dose. As a positive control, mice received a 0.1 mL dose of the commercially available inactivated vaccine (Fel‐O‐Vax PCT, Zoetis). Control mice received LNP‐empty by intramuscular injection. Blood was collected weekly from mice via the retro‐orbital sinus after the prime immunization. The separated sera were used to determine specific neutralizing antibody titers and to evaluate the level of cellular immunity to the vaccine. At the terminal time points, all mice were humanely euthanized using CO_2_ asphyxiation.

### 2.9. Cat Immunization Experiments and Serum Collection

For in vivo assessment of immune responses induced by mRNA vaccines, healthy susceptible Chinese field cats aged 2–3 months were randomly divided into three groups (*n* = 4). One group was administered 20 μg of LNP‐VP1‐mRNA vaccine via intramuscular injection into the quadriceps muscle. The positive control group was intramuscularly injected with one dose of inactivated vaccine, while the cats in the control group were injected with LNP‐empty. The vaccinated cats also received a booster immunization with the same dose 3 weeks after the first immunization. Respectively, blood samples were collected from the jugular vein at weeks 0, 2, 3, 4, 5, and 6 after the primary vaccination, and the serum was analyzed further.

### 2.10. ELISA for Evaluating Serum Antibody Responses

To determine the level of VP1 protein‐specific binding antibody in serum after vaccine immunization, the ELISA plate was pre‐coated with FCV antigen and placed at 4°C overnight. The plate was incubated with PBS containing 5% low‐fat milk powder for 2 h at 37°C, after which the sealing solution was discarded. Then, diluted serum samples (1:100) of 100 μL per well were added to the wells of the plate. After 1.5 h of incubation, the plate was washed, and goat anti‐mouse/cat IgG‐HRP antibody (Easybio, SBA, 1:1000) was added to the wells and incubated for 1 h at 37°C. Afterwards, the plate was washed and incubated with the substrate tetramethylbenzidine (TMB; Beyotime, Shanghai, China) for 10 min at room temperature. Then, the reaction was terminated by adding an equal amount of 2 mol/L sulfuric acid, and then the absorbance was read at 450 nm using the SpectraMax 190 spectrophotometer (Molecular Devices, California, USA). A reading 2.1 times higher than that of the negative control well was considered positive.

### 2.11. Neutralization Assays

Neutralizing antibody titers were determined by a virus neutralization assay [33]. Collected serum samples were heat inactivated for 30 min at 56°C and then diluted 4‐fold from 1:8. The diluted samples were then mixed with the diluted virus (100 TCID_50_) in equal volumes. The mixtures were then incubated at 37°C with 5% CO_2_. After 2 h of reaction of the serum and the virus, the mixture was transferred to 96‐well plates seeded with CRFK cells and cultured at 37°C in 5% CO_2_ for 2 days. The neutralizing antibody titers were calculated based on the cytopathic effect using the Reed–Muench method.

### 2.12. Evaluation of Cellular Immune Response

Cellular immune responses of the FCV VP1‐mRNA vaccine were assessed by measuring IL‐4, IL‐6, IFN‐γ, and TNF‐β levels in mice or cats using precoated ELISA kits (MEIMIAN, Jiangsu, China), according to the manufacturer’s instructions. Briefly, the standard samples were serially diluted and sequentially added to the microtiter plate along with the samples to be tested, taking care not to touch the well walls during addition. The plate was then covered with a membrane, gently shaken to ensure mixing, and incubated for 30 min at 37°C. The plates were washed and incubated with the enzyme‐conjugated working solution for 30 min at room temperature. Finally, the plate was incubated with TMB substrate solution, and after the addition of the stop solution, the OD values of each well were measured at a wavelength of 450 nm using a microplate reader.

### 2.13. Experimental Challenge of Cats With FCV Strain

First, 3 weeks after booster immunization, cats were anesthetized with an intravenous injection of 4 mg/kg propofol and intranasally inoculated with 1 × 10^8.2^ TCID_50_ of an FCV strain suspension (0.5 mL per cat). The survival rate and clinical symptoms of cats were monitored regularly every day for 15 days postinfection, including body temperature, oral ulcers, eye and nose secretions, body weight, and mental state. Regulations of the European Pharmacopeia and relevant literature were used to establish a clinical scoring system for FCV to assess the clinical symptoms of infected cats (Table 1) [1, 34, 35]. The severity levels of the parameters ranged from 0 (none) to 3 (severe) or 5 (death).

**Table 1:** Clinical scoring system used to monitor the clinical evidence of FCV.

Clinical symptoms	Description of clinical symptoms	Score
Body temperature	38–39.5°C	0
	≥39.6°C	1
	≥40 or ≤37°C	2

Body weight	Steady increase	0
	Within 3% reduction	1
	Reduction of 3% and above	2

Oral ulcers	None	0
	Small and few	1
	Big and many	3

Nose secreta	None	0
	Few	1
	Many	2

Eye secreta	None	0
	Few	1
	Many	2

Other symptoms	None	0
	Few	1
	Many	2

State of existence	Survival	0
	Death	5

### 2.14. Clinical Scoring of Cats Following FCV Challenge

Clinical symptoms in FCV‐infected cats were evaluated using an established scoring system based on varying degrees of severity (Table 1). Severity of oral lesions significantly influenced total scores: 0 (none), 1 (1–2 small ulcers <2 mm), or 3 (>3 ulcers or ≥2 mm diameter). Additionally, the scoring system allocated 2 points for each parameter, including abnormal body temperature (≥40 or ≤37°C), ≥3% body weight loss, and increased nasal or ocular secretions. The scoring system allocated 5 points in the event of death. Disease severity across experimental groups was compared using this scoring system.

### 2.15. Statistical Analysis

Data analysis was performed using GraphPad Prism software version 10.0 (GraphPad Software, Inc., CA). Survival statistical analyses were performed using the log‐rank test (Mantel‐Cox). For other data, two‐way ANOVA and post hoc tests were used to analyze statistical differences between groups. The following symbols were used to indicate significant differences between groups:  ^∗^, *p* < 0.05;  ^∗∗^, *p* < 0.01;  ^∗∗∗^, *p* < 0.001;  ^∗∗∗∗^, *p* < 0.0001; ns, not significantly different.

## 3. Results

### 3.1. Design and Construction of the FCV‐VP1 mRNA

We designed an mRNA vaccine candidate encoding a modified FCV‐VP1 capsid protein, with a 124‐amino acid deletion. The mRNA molecule begins with a 5^′^ cap, a SP, and the 5^′^ UTR from human α‐globin, followed by an optimized target antigen VP1 sequence, a 3^′^ UTR from human α‐globin, and ends with a 100‐nucleotide poly(A) tail (Figure 1A). The sequence was linked to the pUC57 cloning vector to construct a recombinant plasmid containing the DNA template for VP1 mRNA transcription. The recombinant plasmid was linearized with *Bsa* I digestion, and the results showed that the size of the digested fragment was consistent with the expected size (Figure 1B, Left). The linearized DNA underwent IVT and capping reactions, resulting in the production of fully mature mRNA that resembles host mRNA. The mRNA product migrated as a single sharp band without signs of degradation by agarose gel (Figure 1B, Right), and capillary electrophoresis analysis confirmed that the constructs maintained full‐length integrity (Figure 1C). In vitro antigen expression was verified by detection of the VP1 proteins in the mRNA‐transfected BHK‐21 cells. The indirect IFA results showed that VP1‐mRNA efficiently expressed FCV VP1 proteins in vitro (Figure 1D). Transfection of BHK‐21 cells with LNP‐VP1‐mRNA resulted in VP1 protein expression in cell lysate (Figure 1E).

Figure 1Design and construction of FCV‐VP1 mRNA. (A) Schematic of the FCV genome and illustration of mRNA constructs. An mRNA was designed to encode the VP1 viral protein with a 124‐amino acid deletion and an N‐terminal signal peptide (SP) sequence, 5^′^ and 3^′^ untranslated regions (UTRs) flanking the coding sequence, a 5^′^ cap, and a 3^′^ poly(A) tail. (B) Linearization of plasmid DNA by restriction enzyme (left). IVT with cap1 capping and purified mRNA nucleic acid electrophoresis (right). (C) The capillary electrophoresis profiles of in vitro synthesized VP1‐mRNA. The electropherogram shows the optimal purity of synthesized mRNA. (D) The expression of mRNA vaccines 24 h after transfection into BHK‐21 cells detected by fluorescence microscopy (green: anti‐VP1, blue: nuclei stained with DAPI). All scale bars = 200×. (E) In vitro synthesized VP1 mRNA was transfected into BHK‐21 cells. Lysate was analyzed by Western blotting with a VP1 polyclonal antibody. Shown are representative blots.

**Figure:**
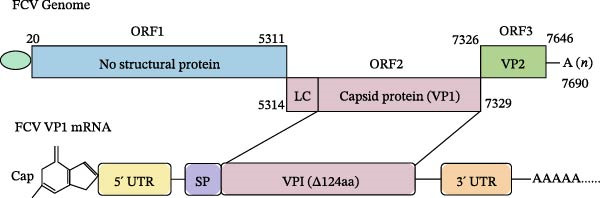
(A)

**Figure:**
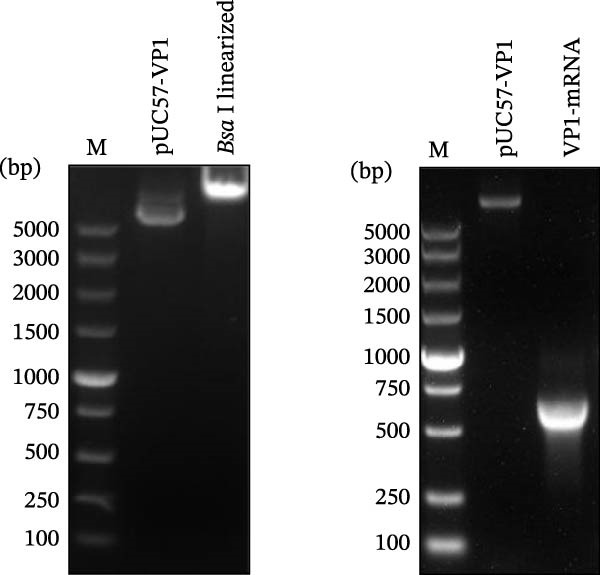
(B)

**Figure:**
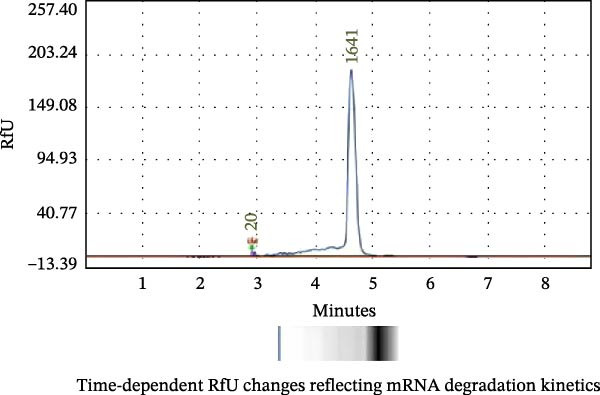
(C)

**Figure:**
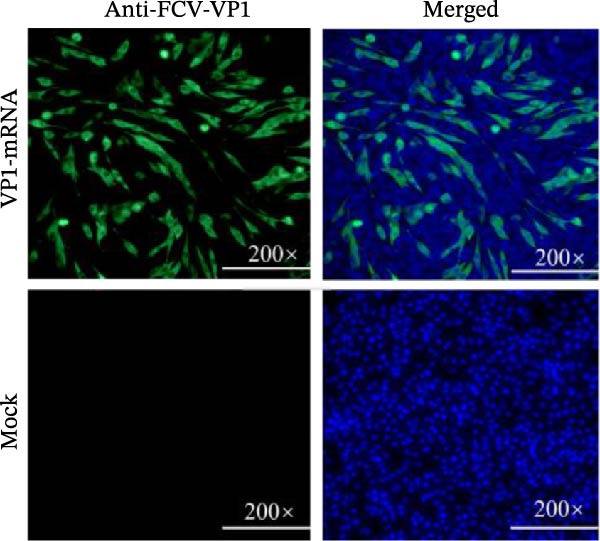
(D)

**Figure:**
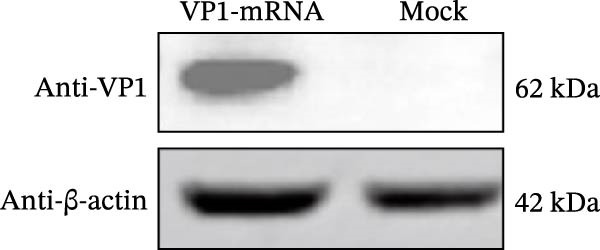
(E)

### 3.2. In Vitro Characterization of LNP‐VP1‐mRNA Vaccine

To achieve highly efficient and prolonged antigen expression by mRNA in vivo, VP1‐mRNA was prepared for vaccination by encapsulation in LNPs, resulting in LNP‐VP1‐mRNA vaccine (Figure 2A). The size distribution and zeta potential of the LNP‐VP1‐mRNA vaccine were measured using a Zetasizer Ultra analyzer. The particle size was 228.4 nm, with a narrow PDI of 0.169 (Figure 2B), and the zeta potential was −1.67 mV (Figure 2C). After the vaccine was prepared, it was stored in a 5% sucrose solution at −80°C for long‐term preservation. To evaluate the stability under practical storage conditions of the LNP‐mRNA vaccine formulation, samples were taken every week to measure particle size, PDI, and zeta potential. Results showed that the vaccine was relatively stable at −20°C for 1 month, with no significant changes in particle size or zeta potential (Figure 2D). The results demonstrate that the LNP‐VP1‐mRNA vaccine exhibits good dispersibility, displaying typical characteristics of LNP formulations.

Figure 2Characterization of the LNP‐VP1‐mRNA vaccine. (A) Schematic diagram of the LNP‐VP1‐mRNA vaccine construct. The mRNA was mixed in an acidic aqueous solution, combined with organic phase lipids, and the mixture was extruded through vortexing into lipid nanoparticles (LNPs). (B) The particle size and polydispersity index (PDI) graph of LNP‐VP1‐mRNA measured using the Zetasizer Ultra instrument analyzer. (C) Zeta potential of the LNP‐VP1‐mRNA vaccine. (D) Stability assessment of the LNP‐VP1‐mRNA vaccine: Changes in particle size and zeta potential of LNP‐mRNA samples stored at −20°C for up to 1 month.

**Figure:**
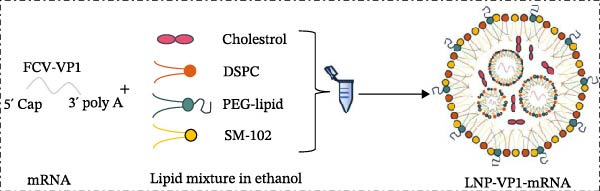
(A)

**Figure:**
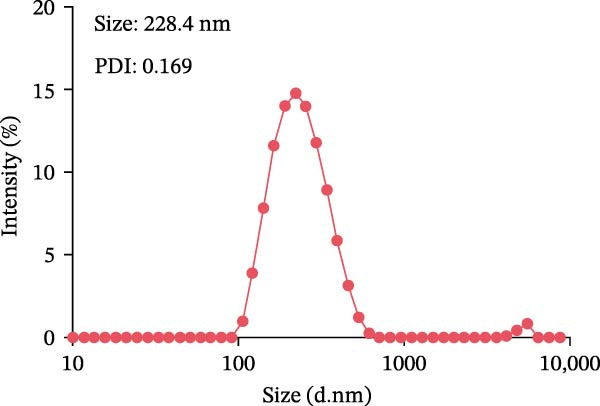
(B)

**Figure:**
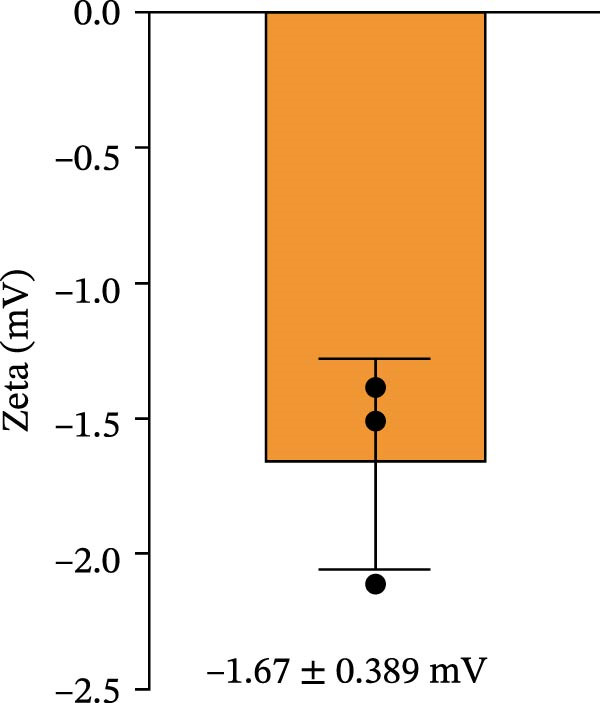
(C)

**Figure:**
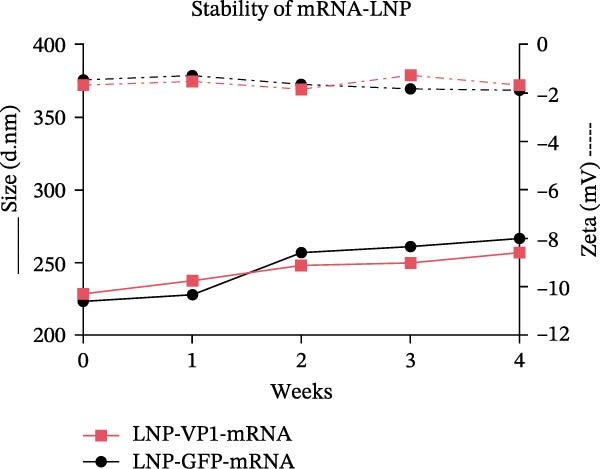
(D)

### 3.3. Immunogenicity of LNP‐VP1‐mRNA Vaccine in Mice

The immune responses induced by LNP‐VP1‐mRNA vaccine candidate were evaluated in mice. BALB/c mice (*n* = 4) were immunized with 5 μg of LNP‐VP1‐mRNA vaccine by intramuscular injections; the same volume of LNP‐empty was immunized as a negative control, and 0.1 dose of inactivated vaccine was included as a positive control, following a prime‐boost immunization schedule (0 and 4th week). Blood samples were collected weekly for anti‐FCV IgG and immunization‐related analyses (Figure 3A). Anti‐FCV IgG levels were measured by ELISA in mice sera collected weekly to assess the duration of antibody persistence following immunization. Anti‐FCV IgG remained detectable at 7 weeks postimmunization, showing a significantly increasing trend similar to the inactivated vaccine group (Figure 3B). Anti‐FCV IgG levels reached their highest concentration 1 week after the booster immunization (Figure 3C). Neutralizing antibody titers were assessed in postimmunization sera. Both the LNP‐VP1‐mRNA vaccine and the inactivated vaccine induced neutralizing antibodies in BALB/c mice, with no significant difference in FCV‐specific neutralizing antibody levels between the two groups (Figure 4A). Neutralizing antibody titers reached their highest concentration 1 week following the booster immunization. No neutralizing antibodies were detected in the LNP‐empty group (Figure 4B). These results demonstrate that LNP‐VP1‐mRNA vaccine induces strong immunogenicity and a highly neutralizing antibody response. To assess cellular immune responses induced by the LNP‐VP1‐mRNA vaccine following priming and boosting, cytokine levels were measured by ELISA. ELISA results showed that LNP‐VP1‐ mRNA vaccinated mice produced significantly higher levels of IFN‐β and IL‐4 compared to control mice (Figure 4C, D). Taken together, these findings demonstrate that the LNP‐VP1‐mRNA vaccine induced a potent humoral immune response and cellular immune responses in mice.

Figure 3Immunogenicity of LNP‐VP1‐mRNA vaccine in mice. (A) Mice were immunized at weeks 0 and 4 via intramuscular injection with 5 μg of LNP‐VP1‐mRNA vaccine, LNP‐empty, and 0.1 dose of inactivated vaccine (*n* = 4). Serum was collected for IgG and immunogenicity testing weekly after the initial vaccination. (B) The levels of anti‐FCV IgG in serum were measured for 7 consecutive weeks by ELISA. (C) The levels of anti‐FCV IgG were measured 1 week after the boost immunization by ELISA. Two‐way ANOVA was used to evaluate intergroup differences. Error bars represent SEM ( ^∗^ *p* < 0.05;  ^∗∗^ *p*  < 0.01;  ^∗∗∗^ *p*  < 0.001;  ^∗∗∗∗^ *p*  < 0.0001; ns, no significant difference).

**Figure:**
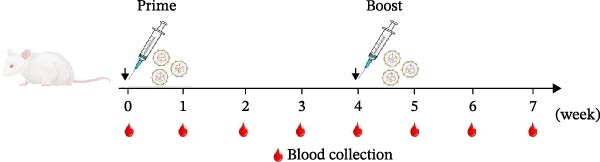
(A)

**Figure:**
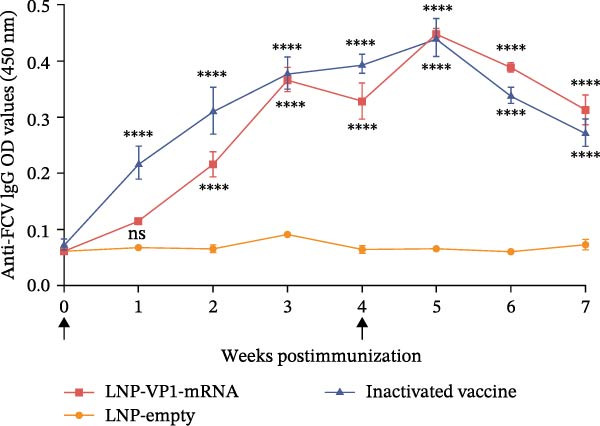
(B)

**Figure:**
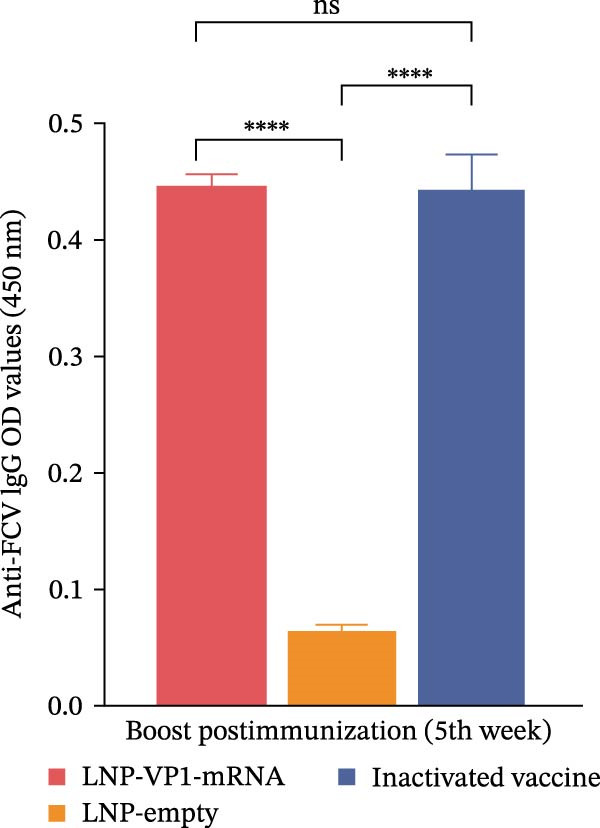
(C)

Figure 4Neutralization activity and cellular immune responses induced by LNP‐VP1‐mRNA vaccine in mice. (A) The neutralizing antibody titers were quantified. (B) Neutralizing antibody titers 1 week after the boost immunization. (C, D) The serum was collected for the detection of IFN‐β (mice) and IL‐4 (mice) by ELISA. Two‐way ANOVA was used to evaluate intergroup differences. Error bars represent SEM ( ^∗^ *p* < 0.05;  ^∗∗^ *p* < 0.01;  ^∗∗∗^ *p* < 0.001;  ^∗∗∗∗^ *p* < 0.0001; ns, no significant difference).

**Figure:**
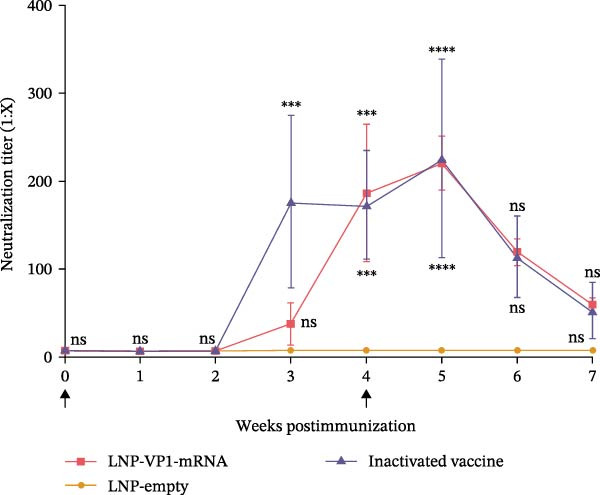
(A)

**Figure:**
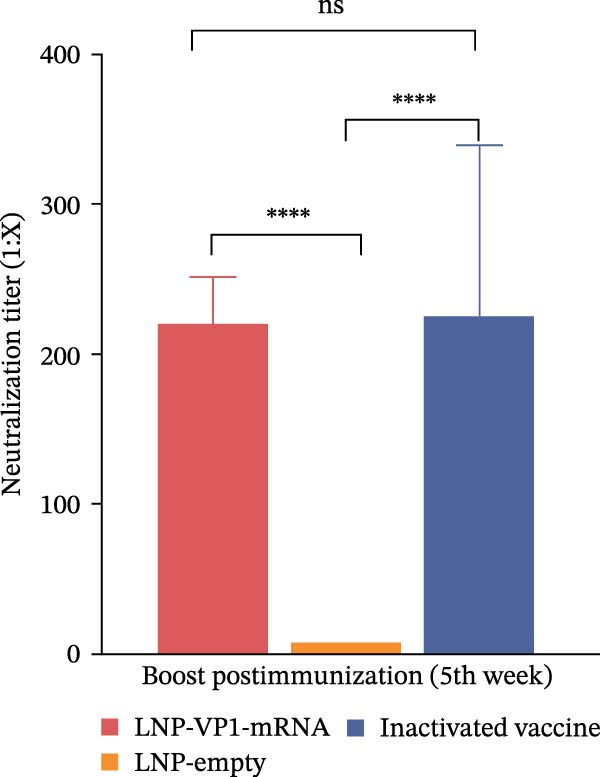
(B)

**Figure:**
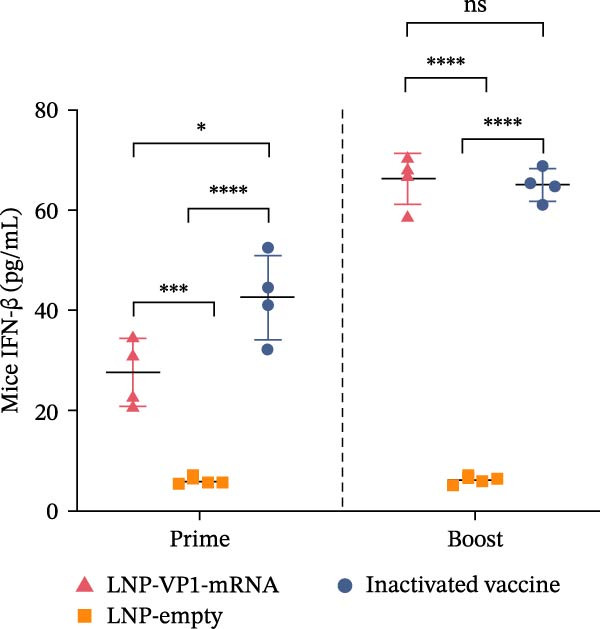
(C)

**Figure:**
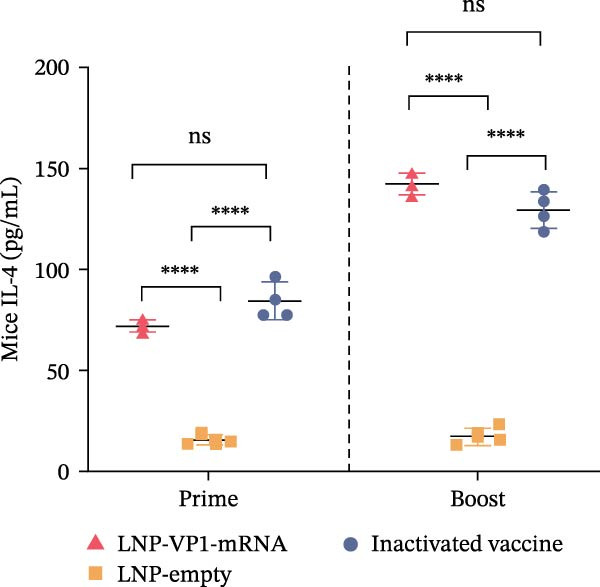
(D)

### 3.4. Rapid Antibody Induction by LNP‐VP1‐mRNA Vaccine in Cats

Next, we evaluated the humoral immune response induced by the FCV‐VP1‐mRNA vaccine in cats, the natural host of FCV. Serum samples were collected before vaccination to evaluate the levels of anti‐FCV IgG and neutralizing antibodies. Cats were administered intramuscular injections with the LNP‐VP1‐mRNA vaccine at 20 μg per cat (*n* = 4). Cats receiving one dose of a commercial inactivated vaccine (Zoetis) or LNP‐empty served as control groups. At 3 weeks postprimary immunization, the cats received the same dose by intramuscular immunization (Figure 5A). We monitored anti‐FCV IgG levels over a 6‐week period. The results showed that the LNP‐VP1‐mRNA vaccine elicited detectable anti‐FCV IgG responses after immunization (Figure 5B), and the highest value was reached 1 week after booster immunization. Additionally, higher anti‐FCV IgG levels were found in cats that received 20 µg of LNP‐VP1‐mRNA vaccine than in those that received a dose of inactivated vaccines (Figure 5C).

Figure 5LNP‐VP1‐mRNA vaccination induces high levels of antibody production in cats. (A) Timeline of LNP‐VP1‐mRNA vaccine immunization and evaluation. Cats were immunized with 20 μg of the LNP‐VP1‐mRNA vaccine, while LNP‐empty served as negative controls (*n* = 4/group). Additionally, cats administered one dose of the inactivated vaccine were included as positive controls. Two intramuscular immunizations were given on weeks 0 and 3. Sera were collected at the indicated time points. (B) The levels of anti‐FCV IgG were measured for 6 consecutive weeks by ELISA. (C) The levels of anti‐FCV IgG in serum were measured 1 week after the boost immunization by ELISA. Two‐way ANOVA was used to evaluate intergroup differences. Error bars represent SEM ( ^∗^ *p* < 0.05;  ^∗∗^ *p* < 0.01;  ^∗∗∗^ *p* < 0.001;  ^∗∗∗∗^ *p* < 0.0001; ns, no significant difference).

**Figure:**
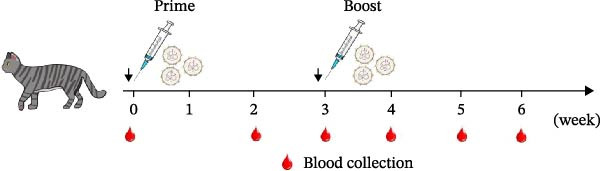
(A)

**Figure:**
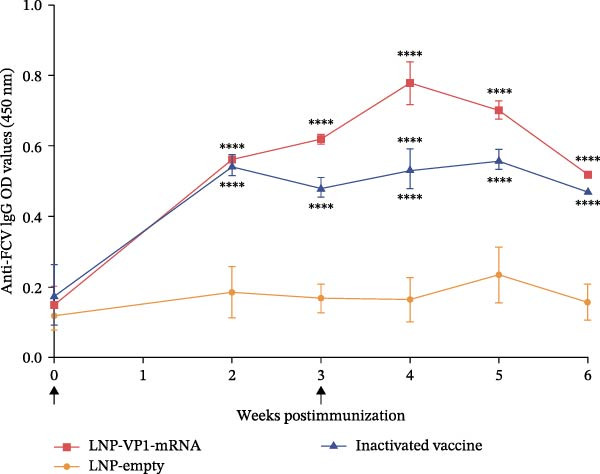
(B)

**Figure:**
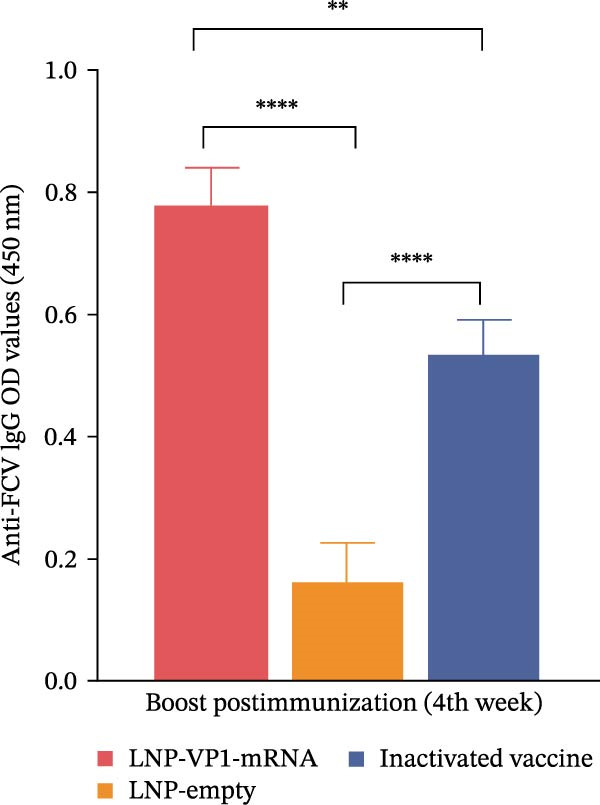
(C)

### 3.5. LNP‐VP1‐mRNA Vaccine Activates Neutralization Activity and Cellular Immune Responses in Cats

Furthermore, the neutralizing antibody titers exhibited a sustained increase after the booster immunization (Figure 6A). Consistent with the results observed in mice, neutralizing antibody levels reached their peak 1 week after the booster immunization (Figure 6B). These results indicate that the LNP‐VP1‐mRNA vaccine elicited a robust humoral immunity in cats. Cellular immunity levels were measured by ELISA 1 week after primary and booster immunizations. The results showed that cats immunized with the LNP‐VP1‐mRNA vaccine and inactivated vaccine induced higher levels of Th1‐related cytokines (IFN‐γ and IFN‐β) and Th2‐related cytokines (IL‐4 and IL‐6) compared to the control group (Figure 6C–F). These results indicate that the LNP‐VP1‐mRNA vaccine effectively stimulates both Th1‐ and Th2‐type cellular immune responses in cats.

Figure 6Neutralization activity and cellular immune responses induced by LNP‐VP1‐mRNA vaccine in cats. (A) The neutralizing antibody titers were quantified. (B) Neutralizing antibody titers 1 week after booster immunization. Detection of IFN‐γ (felines) (C), IFN‐β (felines) (D), IL‐4 (felines) (E), and IL‐6 (felines) (F) was analyzed by ELISA 1 week after primary and booster immunization. Two‐way ANOVA was used to evaluate intergroup differences. Error bars represent SEM ( ^∗^ *p* < 0.05;  ^∗∗^ *p* < 0.01;  ^∗∗∗^ *p* < 0.001;  ^∗∗∗∗^ *p* < 0.0001; ns, no significant difference).

**Figure:**
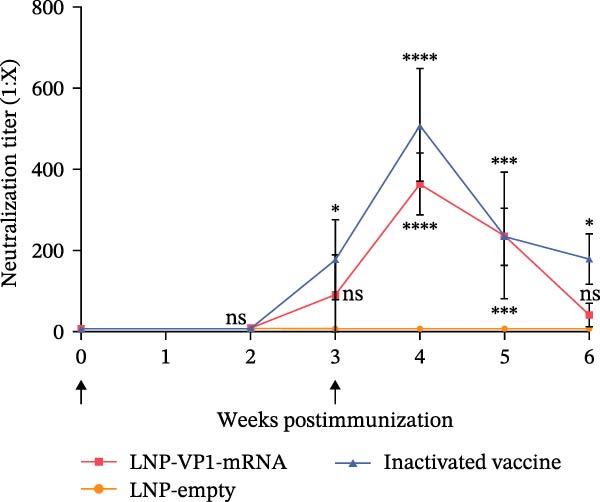
(A)

**Figure:**
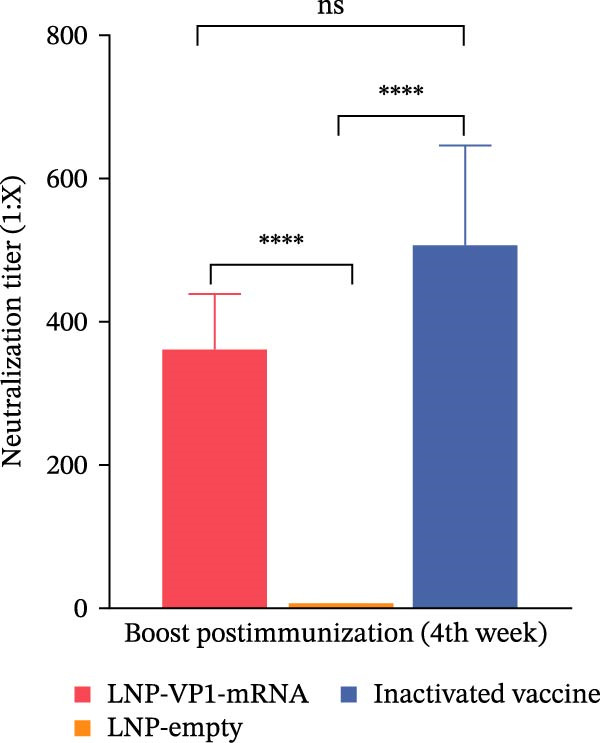
(B)

**Figure:**
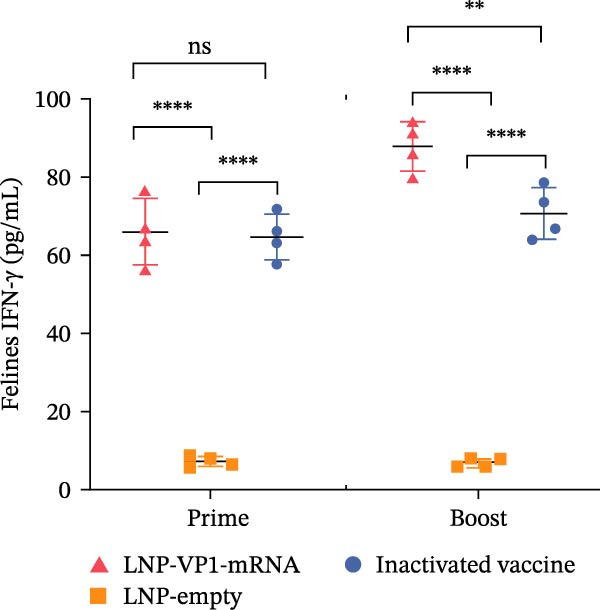
(C)

**Figure:**
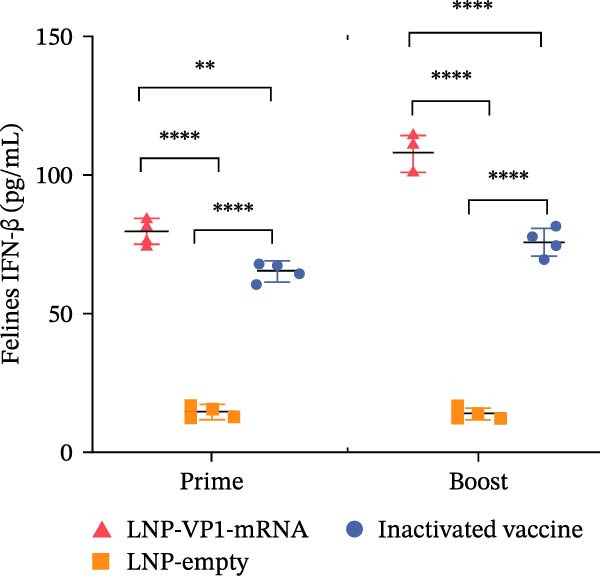
(D)

**Figure:**
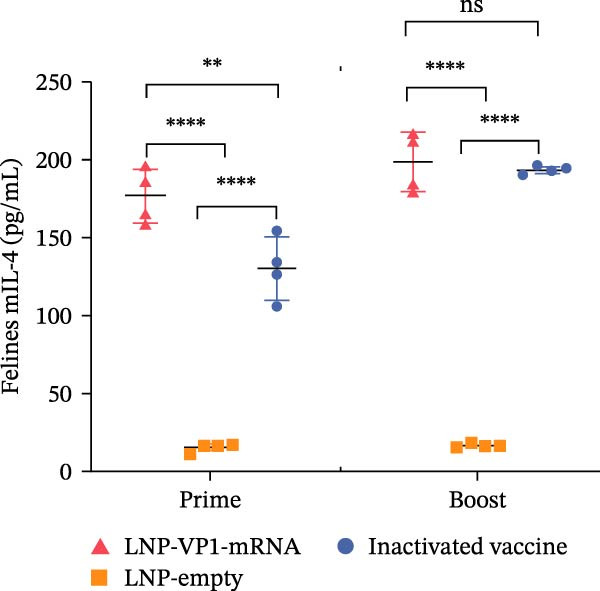
(E)

**Figure:**
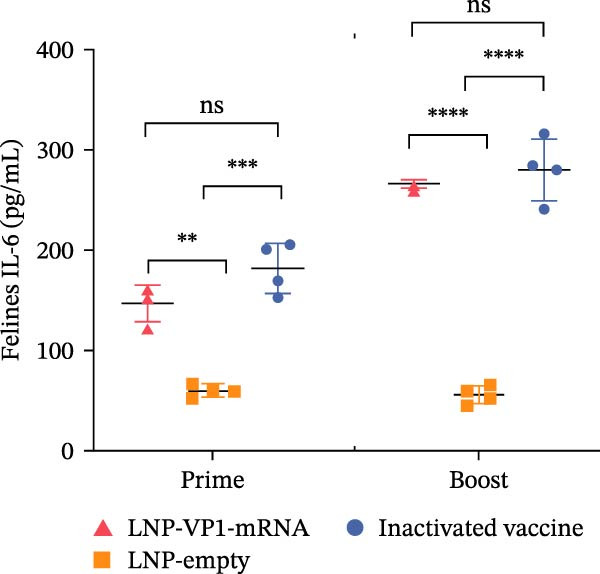
(F)

### 3.6. LNP‐VP1‐mRNA Vaccine Provides Full Protection Against FCV Challenge in Cats

To comprehensively evaluate the protective efficacy of LNP‐VP1‐mRNA vaccine against FCV challenge in vivo, cats (*n* = 4) were randomly allocated to three groups. One group was immunized intramuscularly with 20 μg of LNP‐VP1‐mRNA vaccine, another group received LNP‐empty as a negative control, and the third group was given one dose of inactivated vaccine as a positive control. Three weeks postimmunization, all cats were challenged oronasally with 1 × 10^8.2^ TCID_50_ of the virulent FCV strain in 0.5 mL (Figure 7A). Cats in the LNP‐empty cohort presented overt clinical manifestations consistent with acute FCV infection, characterized by oral ulceration, hypersalivation, lethargy, and prominent lesions involving the oral and ocular mucosae. Conversely, the LNP‐VP1‐mRNA‐vaccinated group remained clinically asymptomatic throughout the observation period, with intact mucosal integrity and unaltered baseline behavior. The inactivated vaccine control group exhibited only mild oral erythema, with no evidence of severe pathological lesions (Figure 7B). The clinical scores monitoring results at various time points post‐challenge showed that there were significant differences in symptom progression among the three experimental groups. In the LNP‐empty group, cats showed typical clinical symptoms of FCV infection. The clinical scores continuously increased after challenge, confirming the successful establishment of the FCV infection model. In contrast, the LNP‐VP1‐mRNA vaccine group maintained significantly lower clinical scores, demonstrating robust protective efficacy against FCV infection (Figure 7C). The survival analysis showed differences among groups. Both LNP‐VP1‐mRNA‐vaccinated cats and the inactivated vaccine control group showed 100% survival within 15 days postinfection. In contrast, the LNP‐empty group showed one FCV‐associated death on Day 11 (Figure 7D). On Day 15 postinfection, cats with persistent clinical signs underwent standardized systemic therapy until clinical signs were fully resolved.

Figure 7The LNP‐VP1‐mRNA vaccine induces and provides full protection against FCV challenge in cats. (A) LNP‐VP1‐mRNA vaccine immunization and FCV challenge program: cats aged 2–3 months were vaccinated with two doses of LNP‐VP1‐mRNA vaccine and LNP‐empty, with one dose as a positive control, administered with an interval of 3 weeks. Then, the cats received FCV strain nasal challenge 6 weeks after the immunization. Clinically observed symptoms were tracked over a period of 15 days. (B) Representative clinical phenotypes following FCV challenge. (C) Clinical symptom scores of tested cats within 15 days postinfection. (D) Survival curve of cats within 15 days postinfection.

**Figure:**
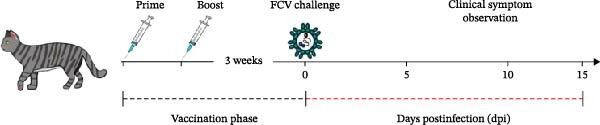
(A)

**Figure:**
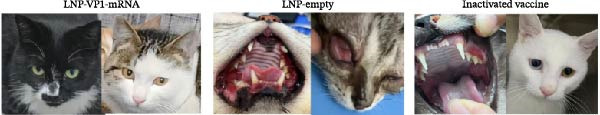
(B)

**Figure:**
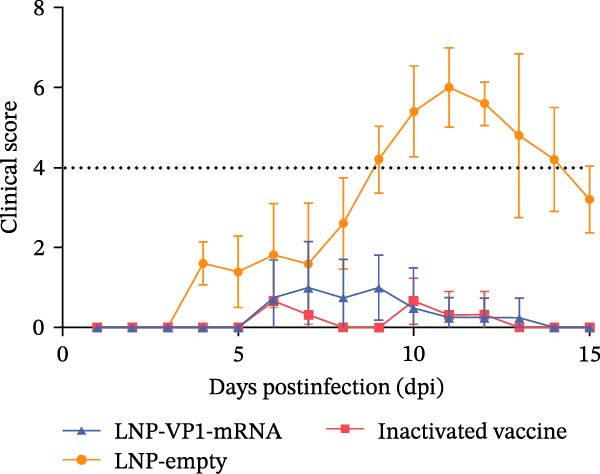
(C)

**Figure:**
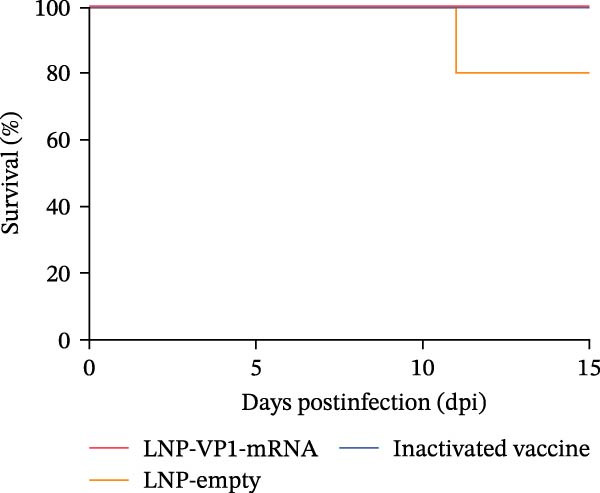
(D)

## 4. Discussion

FCV is a highly mutagenic virus, exhibiting one of the highest evolution rates among known RNA viruses [36]. Currently, vaccination has been the mainstay of control, but the continuous evolution of FCV poses challenges to vaccine design [36]. Several FCV vaccines are currently available, the most common being inactivated vaccines produced by FCV‐F9, FCV‐F255, and FCV‐21, often administered as multiplex vaccines [14, 37]. However, according to the analysis of the prevalence of FCV in southwest China, the neutralizing titer of FCV is low, and the protective effect of existing commercial vaccines against FCV is limited [38]. Therefore, it is crucial to research new vaccines to prevent the disease, mainly involving optimizing antigen design, improving protective efficacy, and increasing the safety of vaccines [39, 40].

The VP1 protein of calicivirus is the most important immune antigen, the main site of interaction between the virus and the host cell. It has most of the neutralizing antibody epitopes, which are key molecules for studying its pathogenicity and developing diagnostic tools [21]. The main capsid protein VP1 is divided into six regions (A–F), each of which has its unique functions and properties [41, 42]. Located at the amino terminus of the protein, the A region is highly conserved and is usually cleaved during capsid protein maturation. Compared with other regions, the C and E regions had the largest variation, among which the high‐variable E region contained most of the neutralizing epitope regions of the virus, which could stimulate the body to produce neutralizing monoclonal antibodies, and the conserved region F had a linear off‐surface recognition of non‐neutralizing antibodies, which could stimulate the body to produce non‐neutralizing monoclonal antibodies [22, 42–45]. Preclinical studies of calicivirus vaccines have shown that the immune protection of the vaccine is related to serum‐neutralizing activity, and neutralizing antibodies have a protective effect against calicivirus infection [46]. Therefore, the VP1 protein was used as the target protein to design the vaccine.

Technical developments in the field of mRNA modification and delivery systems have facilitated the application of mRNA vaccines for the prevention and treatment of infectious diseases [47]. mRNA vaccines have been shown to be safe and immunogenic in humans and animals [48–50]. Whether LNP‐encapsulated mRNA can effectively induce a humoral immune response, especially the high level of neutralizing antibody, is one of the important prerequisites for its protective effect. Previous studies have demonstrated that neutralizing antibodies appeared in the cats from Day 8 to Day 14 after vaccination or infection, with antibody levels differing notably among individual cats [1, 51, 52]. In this study, mice exhibited no difference in induced neutralizing antibody titers compared to commercial vaccines post‐boost immunization. However, in cats, the neutralizing antibody levels induced by commercial vaccines were lower than those induced by the LNP‐VP1‐mRNA vaccine on the 7th day post‐boost immunization. This discrepancy may be attributed to species‐specific immune responses, differences in vaccine formulations, or variations in antigen presentation efficiency between mRNA‐based and inactivated vaccines.

In addition, cellular immunity also has a very important protective effect on humans and animals against viral infections. In this study, the LNP‐VP1‐mRNA vaccine induced significantly higher levels of both Th1‐related (IFN‐γ and IFN‐β) and Th2‐related (IL‐4) cytokines compared to other vaccine groups. Moreover, the IL‐6 level induced by LNP‐VP1‐mRNA was comparable to that in the inactivated vaccine group after the second immunization. Functionally polarized Th cell subsets orchestrate distinct immune pathways: Th1 cells facilitate cellular immunity through macrophage activation and cytotoxic T cell responses, while Th2 cells promote humoral immunity via B cell differentiation and antibody production [53].

The mRNA vaccine was demonstrated to increase the levels of certain cytokines in both mice and cats. Regarding immune protection, following immunization with the VP1‐mRNA vaccine, cats were protected against infection by the FCV strain, exhibiting no noticeable clinical symptoms within 15 days postinfection. This result suggests that the LNP‐VP1‐mRNA vaccine elicits a strong immune response. Furthermore, the mRNA vaccine induces neutralizing antibodies and T cell responses against the challenged FCV strain, and its potential for cross‐protection against other strains warrants further investigation.

In summary, this study successfully designed an mRNA vaccine molecule capable of specifically expressing the calicivirus VP1 protein. The LNP‐VP1‐mRNA vaccine candidate constructed in the study can induce humoral and cellular immune responses in mice and cats, and it can protect cats from FCV infection. This provides insights and a basis for the subsequent development and optimization of FCV vaccines.

## Author Contributions

**Meng-Di Zhang**: writing – original draft, date curation, validation. **Zhuo-Fang Xie, Xin-Hong Li, and Wei Huang**: formal analysis, software, date curation. **Qian-Yu Qian and Hong-Tao Cao**: visualization, investigation, project administration. **Ji-Wei Liu and Ya-Qing Zhang**: formal analysis, conceptualization. **Bin Wang, Yu Qin, and Fu-Shan Shi**: data curation, supervision. **Jian-Bin Tang**: funding acquisition, supervision, visualization. **Yao-Wei Huang**: funding acquisition, supervision. **Yong-Le Yang**: writing – review and editing, funding acquisition, methodology, supervision.

## Funding

This study was supported by the National Natural Science Foundation of China (Grant 32302841). This work was also supported by the Laboratory of Lingnan Modern Agriculture Project (Grant NG2022001), the Specific University Discipline Construction Project (Grant 2023B10564003), and “Pioneer” and “Leading Goose” R&D Program of Zhejiang (Grants 2022C03022 and 2025C01138).

## Conflicts of Interest

The authors declare no conflicts of interest.

## Supporting Information

Additional supporting information can be found online in the Supporting Information section.

## Supporting information


**Supporting Information** Data 1. Full‐length optimized mRNA sequence used in this study.

## Data Availability

All the data generated during the current study are included in the manuscript.
