# Supplementary material for: Engineered VP1 mRNA Vaccine Induces Immunity and Complete Protection Against Feline Calicivirus in Cats
Source: Transbound Emerg Dis. 2026 Feb 6;2026:9499266. doi: 10.1155/tbed/9499266 (PMC12880955; doi:10.1155/tbed/9499266)
Supplement: Supplementary file 1 — Supporting Information Data 1. Full‐length optimized mRNA sequence used in this study. [file TBED-2026-9499266-s001.docx]

**Supporting Material**

Supplementary Data 1. Full-length optimized mRNA sequence used in this study.

Description: The 5' UTR is from position 1 to 57, the codon-optimized VP1 mRNA sequence is from position 64 to 1770, and the 3' UTR is from position 1777 to 1897.

gggaaauaag agagaaaaga agaguaagaa gaaauauaag accccggcgc cgccaccaug 60

ggaacacguc uacauuuuug guggugugga aucuuugcgg uccugaaaua ucugguaugu 120

acuucaagcg cggaugacgg gucaaucaca gcacccgagc aaggaacaau gguuggcggc 180

gucaucgcug aacccagcgc ccagauguca acagcugcug auauggccac cgggaaaagc 240

guugauucug agugggaggc auucuucucc uuucacacca gcgucaauug gaguacaucu 300

gaaacccaag gaaagauucu cuucaaacaa uccuuaggcc cuuugcucaa cccauaucua 360

gaacaccuug cuaagcuaua uguugcgugg ucugggucga uugagguuag guucucuauc 420

ucuggcucug gugucuuugg ugggaagcuc gcagcuauug uuguaccucc ugggguugau 480

ccagugcaga guacuucgau gcuacaauac ccccaugucu uguuugaugc ucgucaggug 540

gaaccaguua ucuucucuau uccugaucua agaagcaccc uguaccaccu uaugucugac 600

acugacacua cauccuuggu cauuauggug uacaaugauc ucaucaaucc cuaugccaau 660

gaugccaacu cuucugggug uauugucacu gucgagacaa aaccuggccc ugacuucaag 720

uuucaccucc uuaagccacc cggaucuaug cuaacccaug guucuguccc uucugauuua 780

auucccaaaa caucuucgcu cuggaucggu aaccgcuacu ggucagacau aacugauuuu 840

gugauucggc cguuugucuu ccaagcaaau cgucauuuug acuuuaauca agagaccgca 900

ggguggagca caccacgguu ucggccuaua ucuguuacca uuagugaaca gaacggagca 960

aaauugggca uuggaguggc aacagauuac auagugccug gaaucccuga uggcuggccu 1020

gacaccacaa uuccugggga guugauacca gcuggcgauu acgcaaucac caaugguacu 1080

ggcaaugaca ucaccacggc uacaggauau gacacugcug auauaauuaa gaacaauacc 1140

aacuuuaggg gcauguacau augugguucg cuccagcgug ccugggguga uaagaaaauu 1200

uccaacacug ccuuuaucac cacugccacc cuagauggug acaacaacaa caagaucaau 1260

cccuguaaua ccauagacca gucaaagauc gucguguuuc aagacaacca uguuggaaag 1320

aaagugcaaa ucucagacga uacauuggcc cugcuugguu acacuggcau uggugagcag 1380

gccaucgggu cugauaggga ccggguugug cgcaucagca cucucccuga aacuggugcu 1440

cgaggcggua accacccaau uuucuacaag aacuccauua aauugggaua uguaauuagg 1500

ucuauugaug ucuuuaauuc acaaaucuug cacacuucca gacaguuauc gcuaaaucau 1560

uaccuacucc cacuugauuc uuuugccguc uauagaauaa uugacucagg uggcucgugg 1620

uuugauauug gaauugauag ugauggguuc ucuuuuguug guguuucugg cuuugguaaa 1680

uuagaauuuc cccuuucugc cuccuacaug ggaauacaau uggcaaagau ccggcuugcc 1740

ucuaacauua ggagucccau gacuaaguua gcuuaagcug gagccucggu ggccuagcuu 1800

cuugccccuu gggccucccc ccagccccuc cuccccuucc ugcacccgua cccccguggu 1860

cuuugaauaa agucugagug ggcggcaaaa aaaaaaaaaa aaaaaaaaaa aaaaaaaaaa 1920

aaaaaaaaaa aaaaaaaaaa aaaaaaaaaa aaaaaaaaaa aaaaaaaaaa aaaaaaaaaa 1980

aaaaaaa 1987
